# Lamb Wave Damage Quantification Using GA-Based LS-SVM

**DOI:** 10.3390/ma10060648

**Published:** 2017-06-12

**Authors:** Fuqiang Sun, Ning Wang, Jingjing He, Xuefei Guan, Jinsong Yang

**Affiliations:** 1 Science and Technology on Reliability and Environmental Engineering Laboratory, Beihang University, Beijing 100191, China; sunfuqiang@buaa.edu.cn (F.S.); wnasd@buaa.edu.cn (N.W.); yangjinsong@buaa.edu.cn (J.Y.); 2 School of Reliability and Systems Engineering, Beihang University, Beijing 100191, China; 3 Siemens Corporation, Corporate Technology, 755 College Rd. E., Princeton, NJ 08540, USA; xuefei.guan@siemens.com

**Keywords:** Lamb wave, GA-based LS-SVM, damage quantification, fatigue crack

## Abstract

Lamb waves have been reported to be an efficient tool for non-destructive evaluations (NDE) for various application scenarios. However, accurate and reliable damage quantification using the Lamb wave method is still a practical challenge, due to the complex underlying mechanism of Lamb wave propagation and damage detection. This paper presents a Lamb wave damage quantification method using a least square support vector machine (LS-SVM) and a genetic algorithm (GA). Three damage sensitive features, namely, normalized amplitude, phase change, and correlation coefficient, were proposed to describe changes of Lamb wave characteristics caused by damage. In view of commonly used data-driven methods, the GA-based LS-SVM model using the proposed three damage sensitive features was implemented to evaluate the crack size. The GA method was adopted to optimize the model parameters. The results of GA-based LS-SVM were validated using coupon test data and lap joint component test data with naturally developed fatigue cracks. Cases of different loading and manufacturer were also included to further verify the robustness of the proposed method for crack quantification.

## 1. Introduction

Guided ultrasonic waves are widely used and have shown great potential in non-destructive evaluations (NDE) and structural health monitoring (SHM) systems. Compared with other guided waves, Lamb waves have the merits of strong penetration, can be used in both isotropic and anisotropic materials, and can interrogate a large area with relatively low energy lost [1]. A large number of studies have reported damage identification using Lamb waves [1,2,3]. Generally, the research of Lamb wave-based damage evaluation can be classified into two groups: damage location identification and damage size quantification. In the former category, identifying and locating an existing damage is the key issue. A considerable number of researchers using signal processing methods and imaging methods, such as tomography [4,5,6,7] and probability-based diagnostic imaging [8,9,10], have focused on this topic. For better diagnosis of the condition of structural health, there is also an increasing interest in more precise damage quantification using Lamb wave methods. The *A*_0_ mode of Lamb wave was employed to identify and locate damages in metallic structures in reference [11]. Ben et al. [12] proposed a Lamb wave propagation method to measure damage location in composite materials. Damage identification was achieved by comparing changes of dispersion characteristics and attenuation between damaged and undamaged carbon fiber reinforced plastic bars. Leong et al. [13] experimentally verified that Lamb wave sensing utilizing scanning laser vibrometry has a potential for effective fatigue crack detection. However, current studies have shown that the proposed method is only valid for cracks longer than 6 mm. He et al. [14] proposed a multi-feature integration method based on a second-order multivariate regression analysis to predict the fatigue crack size. An embedded ultrasonic structural radar algorithm for Lamb wave damage detection was proposed by Victor Giurgiutiu et al. in reference [15,16]. It can effectively identify damages in thin-wall structures by using piezoelectric wafer active sensors (PWAS) phased arrays. Yu et al. [17] employed a sparsely arranged piezoelectric sensor array and developed a focusing array algorithm to quantitatively detect the crack using Lamb wave. Chen et al. [18] proposed a Lamb wave-particle filter-based method for on-line crack propagation prognosis. The particle filter provides the probabilistic results for the crack propagation prognosis. Additionally, the fatigue life prediction is implemented through classical Pairs’ law. Yang et al. [1] employed the Bayesian method to evaluate uncertainties for Lamb wave damage quantification between different targets (such as different geometry and sensor configurations). Due to the complex nature of Lamb wave propagations in virtually infinite combinations of structural shapes and damages, it is highly nontrivial to establish a fully physics-based crack quantification model. In view of this, extensive efforts have been made to develop data-driven based methods. 

Qiu et al. [19] proposed an on-line updating Gaussian mixture model (GMM) for aircraft wing spar damage evaluation under time-varying boundary conditions. Variation indexes of Lamb wave signal and principle component analysis (PCA) were used to obtain the damage sensitive features. A baseline GMM was constructed based on the signal under healthy state. The model is updated over time with new observations to monitor the crack propagation. Legendre et al. [20] presented an ultrasonic non-destructive testing method for welding based on the wavelet transform of Lamb wave signals. A classification process using artificial neural network (ANN) and damage sensitive features extracted from Lamb wave signal was developed to evaluate the welding quality. Su et al. [21] established a Lamb wave-based quantitative identification technique for delamination in composite structures. An ANN model was trained using the spectrographic characteristics extracted from Lamb wave signals to locate the damage. Das et al. [22] presented a method to characterize and classify different damage states in composite laminates by measuring the change in the signature of the Lamb wave that propagates through the anisotropic media under forced excitations. The one-class support vector machine (SVM) was used to perform automatic anomaly detection and damage classification using various features from sensor readings. Both ANN and SVM were used to locate the potential damage site in metallic plates in reference [23]. The study showed that SVM is a robust classifier in the existence of noise and is more computational efficient than ANN. Lu et al. [2] introduced an ANN technique for effective identification of crack damage in aluminum plates with Lamb wave signals, and showed that the diagnostic efficiency and precision are highly dependent on the network architecture. Although a number of studies have been reported to perform Lamb wave-based damage evaluation using data-driven methods, most of them focus on damage identification and classification. It is therefore of significant interest to develop a methodology for reliable, efficient and accurate damage quantification using data-driven models.

This paper presents a Lamb wave-based damage quantification method using a least squares support vector machine (LS-SVM) and genetic algorithm (GA) for metallic materials. The rest of this paper is organized as follows. First, a prognosis framework is introduced based on the Lamb wave and GA-based LS-SVM. The mechanism of Lamb wave damage detection is studied and three damage sensitive features are chosen to train the data-driven model using LS-SVM. The GA method is adopted to optimize the model parameters. Next, coupon tests with different artificial cracks are used to validate the effectiveness of the proposed method. In order to further investigate the accuracy and robustness of the proposed damage quantification method, fatigue testing with naturally developed cracks on lap joint components is performed. Lap-joint specimens are made from two different manufacturers with the same material and geometry and both the constant loading and variable loading case for fatigue testing are also included, in order to verify the robustness of the proposed method. 

## 2. Methodology Development

**Figure 1:**
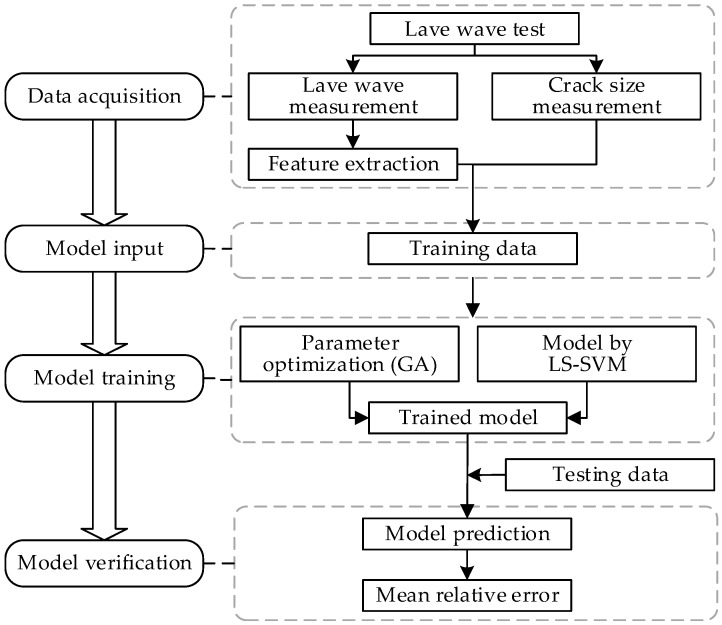
The overall framework of crack size quantitation using Lamb wave and genetic algorithm (GA)-based least square support vector machine (LS-SVM).

The overall framework for Lamb wave damage quantitation using GA-based LS-SVM is illustrated in Figure 1. Firstly, the Lamb wave test is implemented for data acquisition; the damage sensitive features are identified and extracted from the signals as the training data. Next the model parameters are optimized by genetic algorithm and the model is trained based on the learning algorithm of LS-SVM. The resulting GA-based LS-SVM model is validated using both coupon test data and lap joint component test data with naturally developed fatigue crack. In order to further verify the robustness of the proposed method for crack quantification, specimens from different manufactures under different loading spectra are used for fatigue testing. The mean relative error (MRE), which is calculated using the actual and predicted crack size, is used to measure the accuracy of the proposed method. 

### 2.1. Lamb Waves Theory

**Figure 2:**
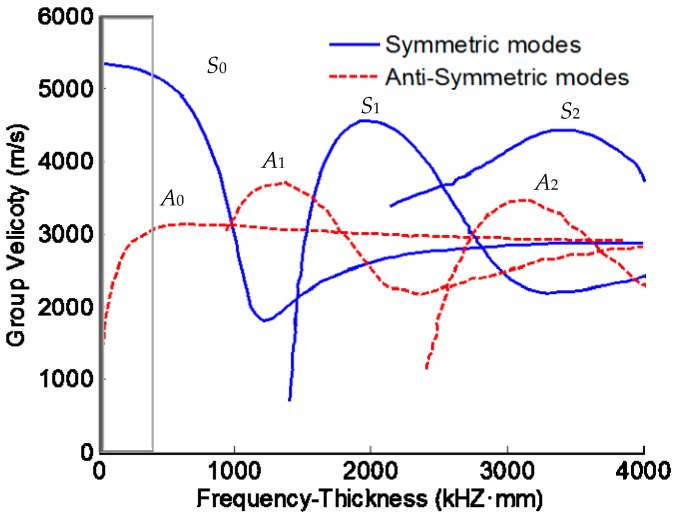
Dispersion curves of Lamb wave of a 2 mm aluminum plate.

**Figure 3:**
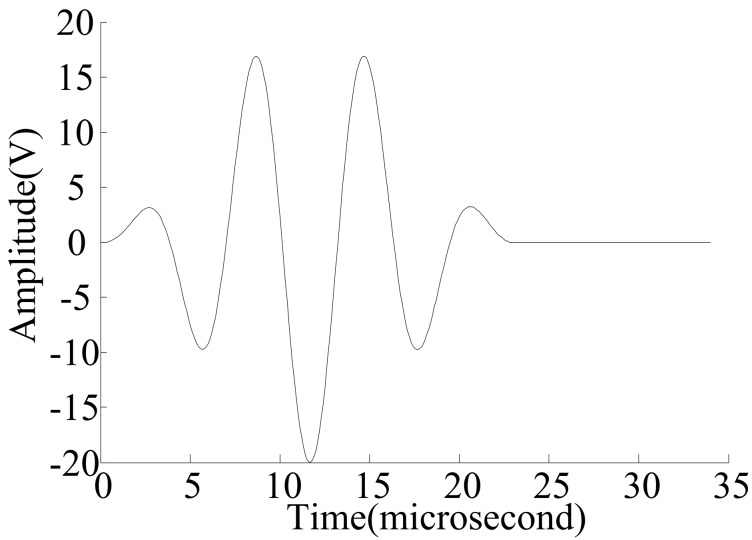
An excitation signal with 0.16 MHz.

As one of the most important guided ultrasonic waves, the Lamb wave is widely used for damage identification. Material discontinuities existing in the wave path can alter wave characteristics (such as energy, wave shape etc.) of the Lamb wave. Thus, monitoring and evaluating the changes of the Lamb wave signal provides a means to analyze damage location and severity. On the other hand, Lamb wave phase velocities are highly dispersed and depend on the product of the frequency and plate thickness. Theoretically, multiple Lamb wave modes exist simultaneously in plate-like structures, and the number of modes increases with the frequency. As can be seen from Figure 2, fewer Lamb wave modes are excited at lower frequencies. Therefore, the response signal is more distinguishable in the low frequency range. It is also known that the lowest-order symmetric mode (*S*_0_) and the lowest-order anti-symmetric mode (*A*_0_) carry more energy and have smaller energy attenuations during propagation, compared to higher-order modes. In addition, the wave length of the *S*_0_ mode (also known as the extensional mode) is significantly larger than the thickness of the plate and it has been proven to be more sensitive to smaller damages than the *A*_0_ mode [9,24,25]. The *S*_0_ mode is used to perform damage quantification in this study. As shown in the shaded area (the product of the frequency and plate thickness is between 0 and 400 kHz·mm) of Figure 2 [25], the group velocity of *S*_0_ is largely non-dispersive with a relative constant group velocity. Given a specimen with a fixed thickness, the actuation frequency is chosen to satisfy this criterion. For example, if the thickness of the specimen is 2 mm, the frequency of the Lamb wave can be set to 0.16 MHz. A symmetrical Hamming-windowed sinusoidal tone bust with 3.5 cycles is used as the excitation in this study, as shown in Figure 3.

**Figure 4:**
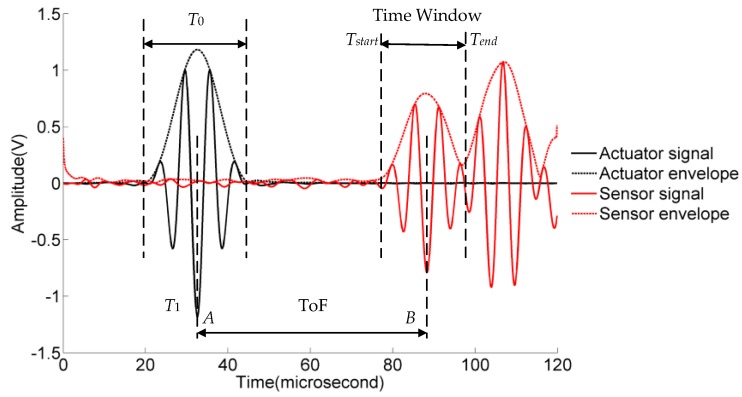
The mechanism of the group velocity and time window calculation.

The presence of material loss, crack or other discontinuities in the path of the Lamb waves can alter the wave characteristics. The energy is weakened due to back-scattering reflection and the transmitted waves are modified due to forward scattering. The detection and quantification of the crack are particularly difficult when the echoes from the specimen boundary and the cracks are superposed. In order to reduce the signal complexity, only the first wave package received by the sensor is used to extract damage sensitive features. To determine the time window for the first wave package, it is essential to calculate the group velocity of the *S*_0_ mode. The group velocity can be estimated by the time-of-flight (ToF) between A and B as shown in Figure 4 with a known wave propagating distance. The Hilbert transform is used to calculate the envelope of the signal. The time window of the first wave package received by the sensor is illustrated in Figure 4 and can be calculated by the following equations

(1) $$T_{start}=T_{1}+ToF-\frac{1}{2}T_{0},$$

(2) $$T_{end}=T_{1}+ToF+\frac{1}{2}T_{0},$$

where *T_start_* and *T_end_* represent the start and stop time point of the time window respectively, *T*_1_ is the time point when the Lamb wave is transmitted, and *T*_0_ is the period time of excitation wave. 

Lamb waves are inherently of a dispersive nature. Therefore the received wave package may vary from the wave package generated by the actuator at the beginning. The impact of this characteristic is not significant in this study due to the short wave transmission distance between actuators and sensors. Moreover, the damage sensitive features from the healthy specimen are used as baseline data to establish the crack length model. The purpose of using a time window is to avoid complex reflection waves from the boundary. Because comparison is made between the baseline and the actual cases, the time window can ensure that the approximately same amount of wave package data is retained in the time window; therefore the time window is not required to include the entire first wave package received by the sensor.

**Figure 5:**
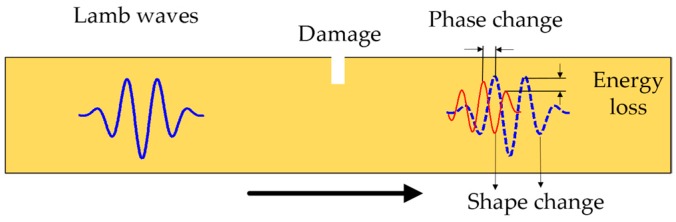
The illustration of the Lamb wave traveling through a damaged region.

By comparing the response signal under damage state and healthy state, it is known that the features of the received signal are different. Figure 5 shows the mechanism of Lamb wave propagation through a damaged region. The crack-like damage can cause backscatter echoes and the energy loss can increase with the increasing of the crack size. On the other hand, the presence of a crack can alter the wave path. For a closed or a partially closed fatigue crack, the received signal contains two parts: directly transmitted wave signals across the crack and echoes from the crack tip. For a notch or a fully opened crack (e.g., a through-thickness crack), the received signals are the waves in a detour route from the crack tip [26]. Based on the above consideration, three damage features, namely, normalized amplitude, phase change, and correlation coefficient, are extracted from the received signal quantitatively as the damage features. In order to eliminate uncertainties from different piezoelectric (PZT) wafers, the normalized amplitude is used as a damage sensitive feature. First, the Hilbert transform is applied for both the received first Lamb wave package and the excitation wave package. The normalized amplitude is obtained by dividing the peak value of the two processed wave packages. The phase change is calculated by subtracting the time of the peak of the damaged signal from that of the healthy signal. The correlation coefficient is calculated by comparing the intact specimen (with 0 mm crack) with the defective specimen in the desired time window calculated based on Equations (1) and (2).

### 2.2. Least Squares Support Vector Machine

**Figure 6:**
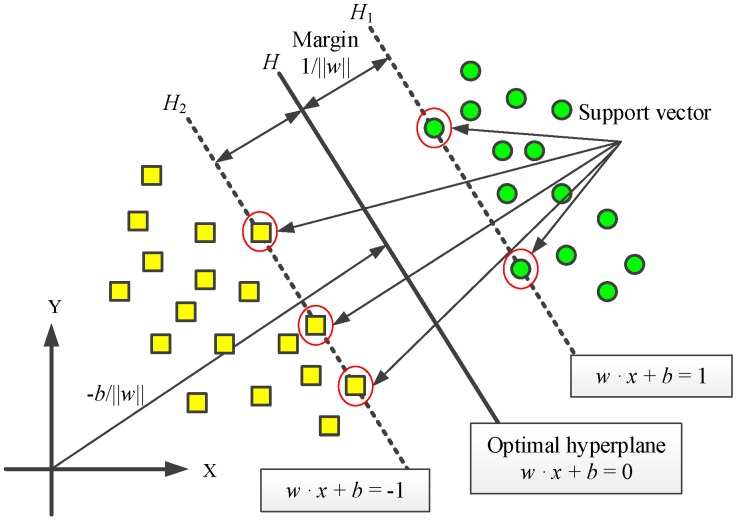
SVM for linear classification.

The support vector machine, a data-driven approach based on statistical learning theory (SLT), was first proposed by Vapnik et al. [27]. Figure 6 shows the fundamental principle of SVM for linear classification, which is an optimization problem of a hyperplane decision boundary. There exist several separating hyperplanes that separate the data of the two classes (data depicted by yellow rectangle and green circle) [28]. Suppose a given training set *D* = {(*x_i_*, *y_i_*) *I* = 1, 2, …, *n*} with input data *x_i_*∈*R^n^* and class labels *y_i_*∈{+1,−1} can be separated without error by a hyperplane *H*: *w*·*x* + *b* = 0, and the lines *|w*·*x* + *b*| = 1 are the boundaries for classification. To obtain better classification accuracy and generalization ability, the margin between the two boundary lines, i.e.,2/||*w*||, is maximized. In addition to classification, the SVM method can be used for regression problems using the so-called *ε*-insensitive loss [27]. 

**Figure 7:**
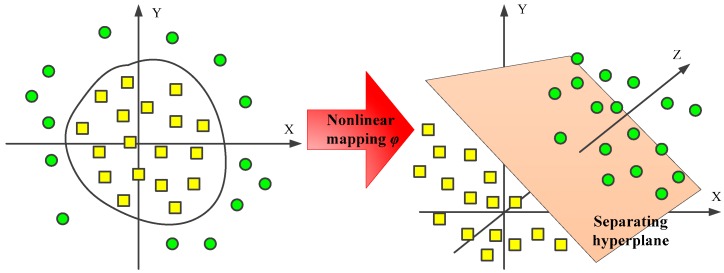
The nonlinear mapping from the input space to a high-dimensional feature space.

For nonlinear classification and regression problems, the input data are mapped to another linearly separable space using a nonlinear function *φ* and the normal linear SVM is applied [29]. The concept is illustrated in Figure 7, where the axes are used to define the spatial dimension.

The least squares support vector machine (LS-SVM) is an improved variant of SVM. It can increase the convergence rate for complex problems [28]. The brief introduction of LS-SVM nonlinear regression theory is briefly reviewed for completeness purposes. Consider a given training data set ${S=\{(x_{i},y_{i}),x_{i}\in R^{n},y_{i}\in R\}}_{n=1}^{N}$, where ***x****_i_* represents the *i*th input vector, *y_i_* is the regression target value corresponding to ***x****_i_*, and *N* is the sample size. The LS-SVM regression model in the primal weight space can be expressed as

(3) $$y\left(x\right)=w^{T}\phi \left(x\right)+b.$$

The term *φ*(·) is a nonlinear mapping function, *w* ∈ *R^n^* and *b* ∈ *R* are model parameters. The associated optimization problem can be formulated as

(4) $$\begin{array}{l} \mathrm{Min}J\left(w,b,e\right)=\frac{1}{2}w^{T}w+\frac{\gamma }{2}\sum_{i=1}^{N}{e_{i}}^{2}=E_{W}+\gamma \cdot E_{D},\\ s.t.e_{i}=y_{i}-\left(w^{T}\phi \left(x_{i}\right)+b\right),i=1,2,...,N, \end{array}$$

where *E_W_* = (1/2)***w****^T^**w***, *E_D_* = (1/2)∑*e_i_*^2^, *γ* is the regularization parameter (also called penalty factor), and *e_i_* represents the prediction error term. The method of Lagrange Multiplier is used to solve the constrained optimization problem, and the Lagrange function is constructed as

(5) $$L\left(w,b,e,\alpha \right)=J\left(w,b,e\right)-\sum_{i=1}^{N}\alpha_{i}\left[w^{T}\phi \left(x_{i}\right)+b+e_{i}-y_{i}\right].$$

where, *α_i_*, *I* = 1, …, *N*, are the introduced Lagrange multipliers. The set of partial derivatives reads,

(6) $$\left\{ \begin{array}{l} \frac{\partial L}{\partial w}=0\Rightarrow w=\sum_{i=1}^{N}\alpha_{i}\phi \left(x_{i}\right)\\ \frac{\partial L}{\partial b}=0\Rightarrow \sum_{i=1}^{N}\alpha_{i}=0\\ \frac{\partial L}{\partial e_{i}}=0\Rightarrow \alpha_{i}=\gamma e_{i}\\ \frac{\partial L}{\partial \alpha_{i}}=0\Rightarrow w^{T}\phi \left(x_{i}\right)+b+e_{i}-y_{i}=0 \end{array}\right.,i=1,2,...,N.$$

After eliminating ***w*** and ***e***, the following linear Karush-Kuhn-Tucker system in ***α*** and *b* is obtained

(7) $$\left[\begin{array}{cc} 0 & {\vec{1}}^{T}\\ \vec{1} & \Omega +\gamma^{-1}I \end{array}\right]\left[\begin{array}{c} b\\ \alpha \end{array}\right]=\left[\begin{array}{c} 0\\ y \end{array}\right],$$

where $\vec{1}$ = [1,…,1]^T^, ***α*** = [*α_1_,…,α_N_*]*^T^*, and ***y*** = [*y_1_,…,y_N_*]*^T^*.

According to the Mercer Theorem, the dot product of *φ*(*x*) can be expressed as a kernel function

Ω*_ij_* = *φ*(***x****_i_*)^T^*φ*(***x****_j_*) = *K*(***x****_i_*, ***x****_j_*), *i*, *j* = 1, …, *N.* (8)

where, *K*(***x****_i_*, ***x****_j_*) is called the kernel function. 

The regression problem (3) can be solved in the dual space of the Lagrange multipliers after applying this kernel trick. Equation (3) can be represented as

(9) $$y\left(x\right)=\sum_{i=1}^{N}\alpha_{i}K\left(x,x_{i}\right)+b.$$

Due to the good generalization ability and fast convergence speed, the following radial basis function (RBF) is used as the kernel function in this paper

(10) $$K\left(x,x_{i}\right)=\exp\left(-\frac{∥x-x_{i}{∥}^{2}}{\sigma^{2}}\right),$$

where *σ*^2^ is the kernel bandwidth parameter, which controls the radical range of the function. 

To construct the LS-SVM regression model with a RBF kernel, it is necessary to select two appropriate tuning parameters: regularization parameter *γ* and RBF parameter *σ*^2^ [28]. Here, the *γ* determines the trade-off between the training error minimization and the smoothness. In practical applications both cross validation and enumeration methods are often adopted to determine the tuning parameters, which require a large computational demand. To alleviate the computational demand, the genetic algorithm is employed to perform the tuning parameters-optimization for LS-SVM regression in the current study.

The genetic algorithm is a method designed for optimization of search problems. It repeatedly modifies the population of individual solutions with an iterative process. The population in each iteration is called a generation. The GA follows three main rules to produce the next generation from the current population [30]. (1) Selection: select the individuals, called parents, which contribute to the population at the next generation; (2) Crossover: combine two parents to form children for the next generation; (3) Mutation: apply random changes to individual parents to form children. In each generation, the fitness function of every individual, which is usually the objective of the optimization problems, is evaluated. Generally, the GA terminates when either a maximum number of generations has been produced or a satisfactory fitness level has been reached.

**Figure 8:**
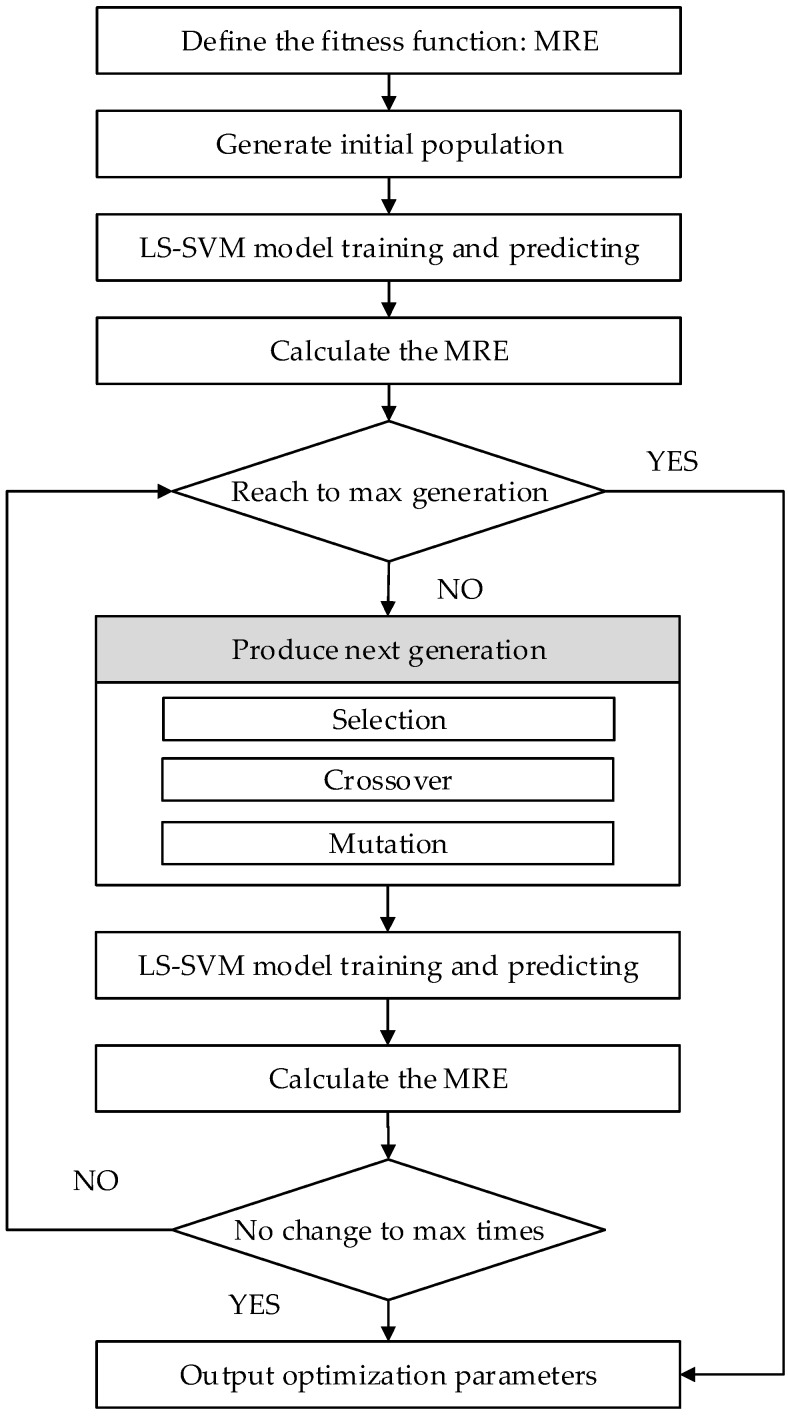
The flowchart of GA optimization for LS-SVM model parameters. MRE = mean relative error.

To optimize the tuning parameters of LS-SVM, the mean relative error (MRE) of the LS-SVM predictors is defined as the fitness function of GA. The MRE can be expressed as

(11) $$MRE=\frac{1}{N}\sum_{i=1}^{N}\frac{\left|y_{i}-\hat{y}\right|}{y_{i}}\times 100\%,$$

where *N* is the sample size, *y_i_* represents the actual value, and $\hat{y}$ is the prediction data. The flowchart of GA based LS-SVM is shown in Figure 8.

## 3. Methodology Validation I: Coupon Test

A simple coupon test is performed in order to verify the efficiency and effectiveness of the proposed method. A pitch-catch sensor configuration is used to perform the damage detection. The normalized amplitude, phase change, and correlation coefficient are extracted from the received signal by signal processing techniques. After that, both the crack size and damage sensitive feature data are used to train the LS-SVM model. The model parameters are optimized by GA, with the optimization objective of minimizing the MRE between actual crack size and prediction data. Additionally, the trained GA based LS-SVM model is employed to predict the crack size using a different data set for validation purposes. 

### 3.1. Experiment

**Figure 9:**
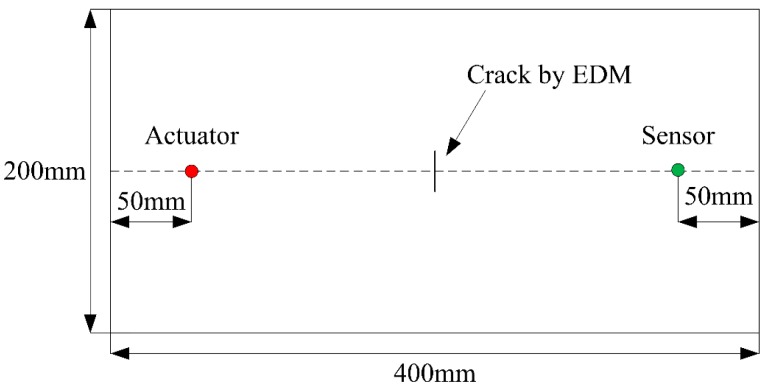
The geometry of the testing specimen and the layout of the piezoelectric (PZT) sensors. EDM = electric discharge machining.

The specimen of coupon test is made of 2024-T3 aluminum. In the center of each specimen, electric discharge machining (EDM) is used to produce a crack with a width of 0.3 mm. The geometry and mechanical properties of the test specimens are shown in Figure 9 and Table 1 respectively. As shown in Figure 9, two piezoelectric (PZT) sensors are placed at each side of the crack as a pitch-catch configuration. The red dot represents the actuator which is used to excite the Lamb wave and the green dot represents the sensor that is used to acquire the Lamb wave. Detailed information of the PZT is shown in Table 2.

**Figure 10:**
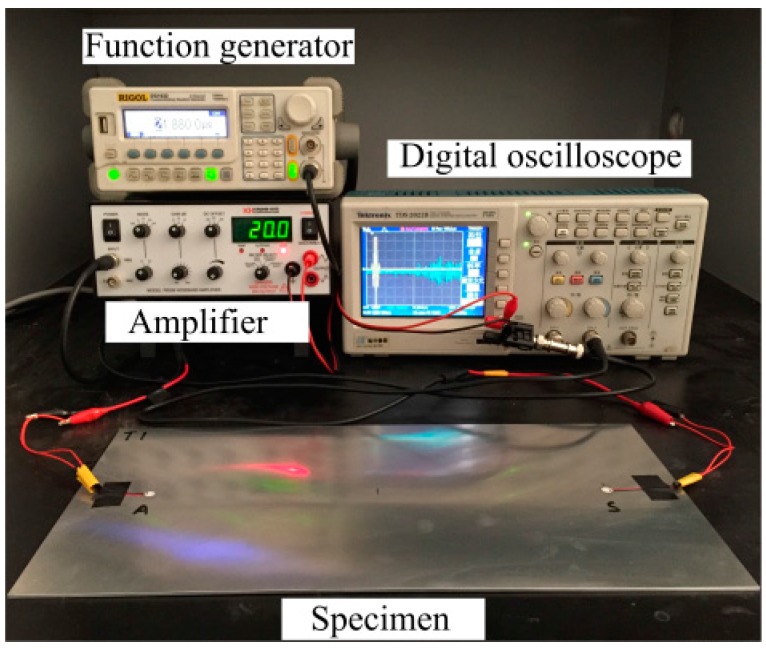
The testing equipment for the coupon test.

**Figure 11:**
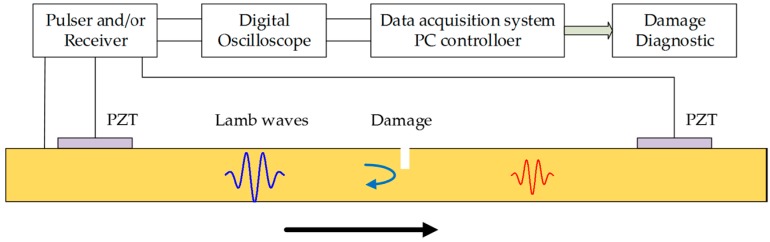
Experimental setup for the damage detection technique.

A multi-channel digital oscilloscope with a sampling frequency of 1 GHz and a resolution of 12 bits is used for Lamb wave acquisition. A total of six specimens are employed for the coupon test and the crack size varies from 2 mm to 20 mm with an increment of 3 mm and from 20 mm to 30 mm with an increment of 5 mm for each specimen. Both the healthy and damaged states are tested respectively. The overall experimental setup is shown in Figure 10 and Figure 11.

The frequency of the Lamb wave is set to 0.16 MHz with a 2 mm thickness specimen to obtain the desired *S*_0_ mode. A symmetrical Hamming-windowed sinusoidal tone bust with 3.5 cycles is used as the excitation. The testing data are collected and the desired time window is obtained based on the procedure described in Section 2.1. 

The extracted damage sensitive features of specimen T1 are shown in Table 3. With increasing crack size, the normalized amplitude and correlation coefficient decrease, and the phase change increases. The results are consistent with the previous discussion, indicating that the selected damage sensitive features are appropriate for the crack size qualification.

**Figure 12:**
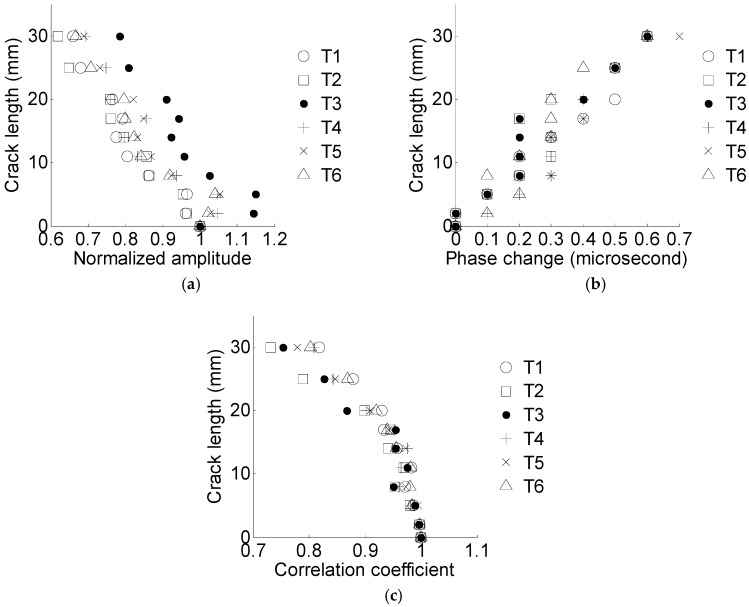
Crack length versus the damage sensitive features for six specimens. (**a**) Crack length versus the normalized amplitude; (**b**) crack length versus the phase change; (**c**) crack length versus the correlation coefficient.

The crack length versus the normalized amplitude, phase change, and the correlation coefficient are shown in Figure 12.

### 3.2. Crack Evaluation Using GA Based LS-SVM

In order to establish the relationship between crack size and damage sensitive features, the GA-based LS-SVM is established and trained using the acquired data. The datasets of T4, T5, and T6 are used for training and the datasets of T1, T2, and T3 are used for validation to verify the effectiveness of the trained model. For the input and output of the LS-SVM write

(12) $$S_{i}=\left[\begin{array}{c} a_{i,1}\\ a_{i,2}\\ \vdots \\ a_{i,10} \end{array}\begin{array}{c} p_{i,1}\\ p_{i,2}\\ \vdots \\ p_{i,10} \end{array}\begin{array}{c} c_{i,1}\\ c_{i,2}\\ \vdots \\ c_{i,10} \end{array}\right],$$

(13) $$L_{i}={\left[\begin{array}{cccc} l_{i,1} & l_{i,2} & \cdots & l_{i,10} \end{array}\right]}^{T},$$

where *S_i_* and *L_i_* are the damage sensitive features and crack size, respectively, for the *i*th specimen. Terms *a_i,j_*, *p_i,j_*, *c_i,j_,* and *l_i,j_* are the normalized amplitude, phase change, correlation coefficient, and crack size, respectively, for the *i*th specimen in the *j*th crack size configuration, *i* = 1, 2, …, 6, *j* = 1, 2, …, 10. Denote *x_train_* as the independent variables of the training data and *y_train_* as the regression target value, the training uses the following set of data

(14) $$x_{train}=\left[\begin{array}{c} S_{4}\\ S_{5}\\ S_{6} \end{array}\right],\;y_{train}=\left[\begin{array}{c} L_{4}\\ L_{5}\\ L_{6} \end{array}\right].$$

**Figure 13:**
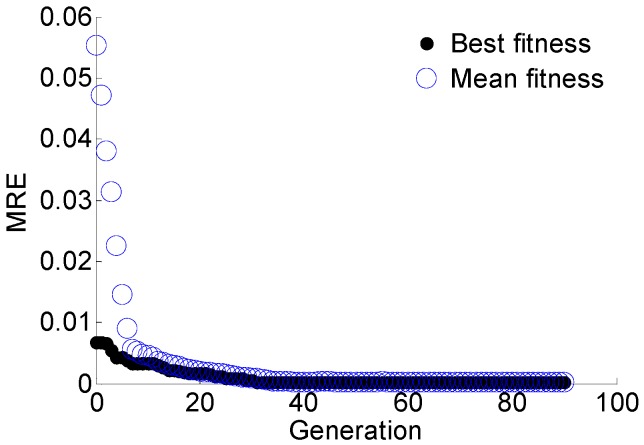
The optimization process using GA for model parameters.

**Figure 14:**
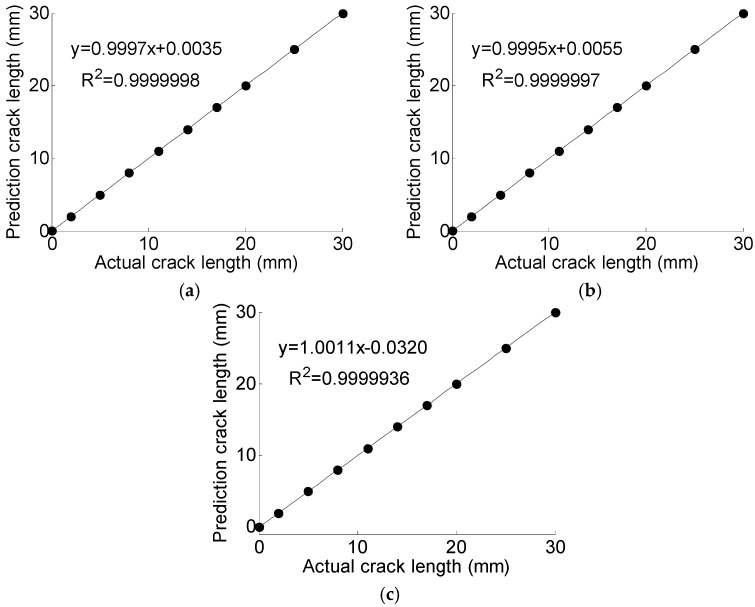
The prediction results using GA based LS-SVM. (**a**) T1; (**b**) T2; (**c**) T3.

The regularization parameter *γ* and the RBF parameter *σ* are optimized using GA with the objective of minimizing the MRE of predication. The optimal target value, i.e., the MRE of predication converges to 0.024%, as shown in Figure 13. In each generation of GA, 50 pairs of parameters are used to train the LS-SVM and calculate the MRE in parallel. The mean fitness is the mean MRE of the total 50 results. According to the optimal results, *γ* = 788.2670 and *σ*^2^ = 0.1050. Using the optimal parameters obtained with GA and the training data, the LS-SVM model is trained for crack size prediction. The prediction results of T1, T2, and T3 are shown in Figure 14. The predicted crack size is represented in the form of a scatter plot. The performance of the proposed GA-based LS-SVM Lamb wave damage quantification model is evaluated by the coefficient of determination *R*^2^. It is observed that the slope of *y= a*_0_*x + a*_1_ in the scatter plots is very close to 1, indicating an accurate prediction. 

**Figure 15:**
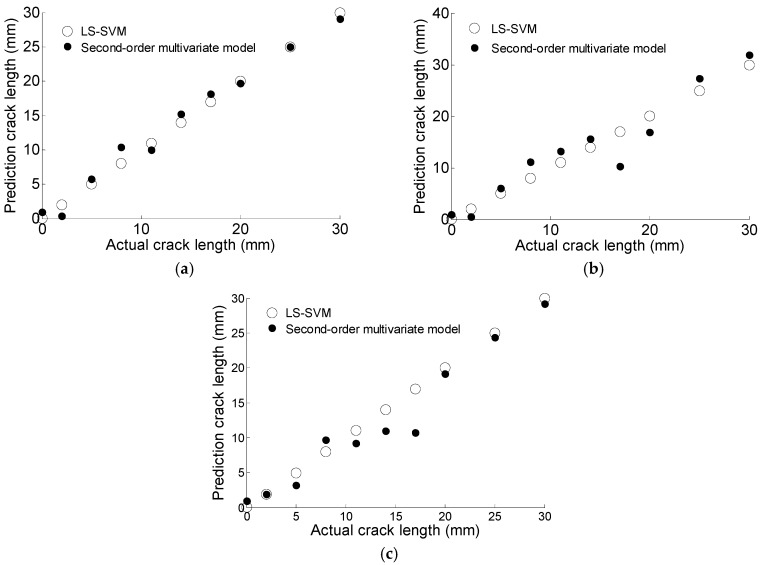
The comparison of prediction data based on second-order multivariate model and GA based LS-SVM. (**a**) T1; (**b**) T2; (**c**) T3.

In [14], a second-order multivariate model is proposed to predict the crack size based on three damage sensitive features mentioned above; the model is given as

(15) $$a=A+\alpha_{1}x+\alpha_{2}y+\alpha_{3}z+\alpha_{4}xy+\alpha_{5}xz+\alpha_{6}yz+\alpha_{7}x^{2}+\alpha_{8}y^{2}+\alpha_{9}z^{2}$$

where *a* is the crack length, *x* is the correlation coefficient, *y* is the phase change, and *z* is the amplitude change. The model parameters are estimated based on regression analysis with experimental data. The regression parameters are shown in Table 4. The prediction results obtained from Equation (15) are compared with that of the GA based LS-SVM model, as illustrated in Figure 15. Results of MRE are shown in Table 5. In this case the GA-based LS-SVM performs better than the alternative method.

### 3.3. Cross Validation

The stability and robustness of the proposed GA-based LS-SVM model is further validated using the rigorous cross validations. The dataset is partitioned into two subsets. One subset is used as training and the other is used for validation. The previous training and validation process is applied. Using the data of T1–T6, it is to be noted that three from six results are chosen, in total 20 different partitions, e.g., C(6,3) = 20. Except for one combination shown previously for methodology illustration, the results of the rest of the 19 combinations are shown in Table 6. An overall satisfactory performance in terms of MRE is observed, indicating that the proposed GA based LS-SVM model can reliably and accurately predict the crack size.

## 4. Methodology Validation II: Lap Joint Components Fatigue Test 

In Section 3, the coupon test was performed to prove the effectiveness of the proposed method. In this section, data from the realistic lap joint components fatigue test with a naturally developed fatigue crack is used to further validate the GA-based LS-SVM damage quantification model.

### 4.1. Experiment

**Figure 16:**
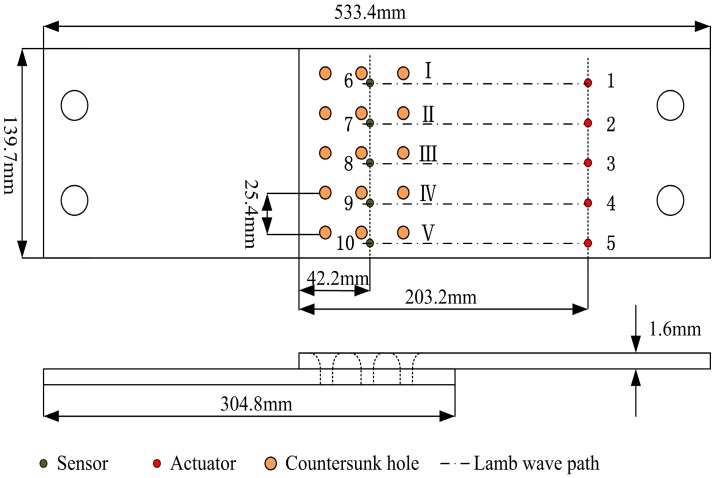
The geometry of the lap joint component and the sensor layout.

**Figure 17:**
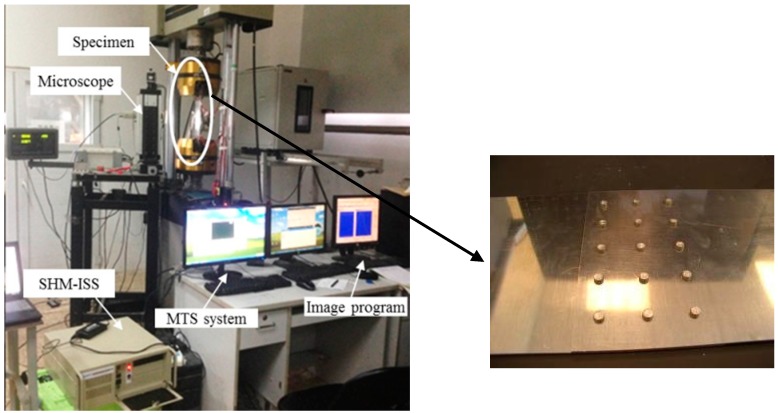
The fatigue crack experimental set up and the lap joint component.

The specimen of lap joint components fatigue test is made of 2024-T3 aluminum with a thickness of 1.6 mm. The mechanical properties of the test specimens are shown in Table 1. The geometry of the testing specimen and the PZT sensor layout are shown in Figure 16. The specimens are three rivet rows by five rivets wide lap joints, consisting of two aluminum panels. The experiment setup and specimens are shown in Figure 17.

**Figure 18:**
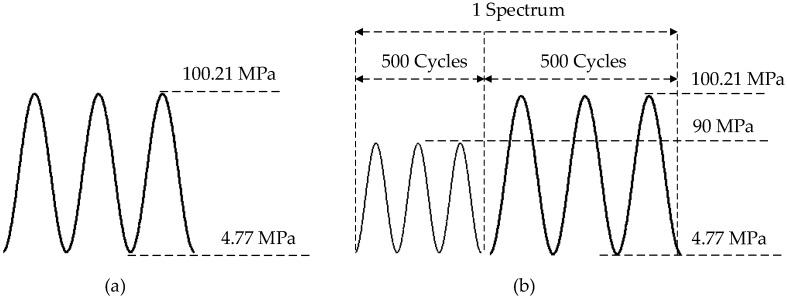
Fatigue loading spectra for lap joint components fatigue test. (**a**) Constant loading spectra for S1–S6; (**b**) variable loading spectra for S7.

**Figure 19:**
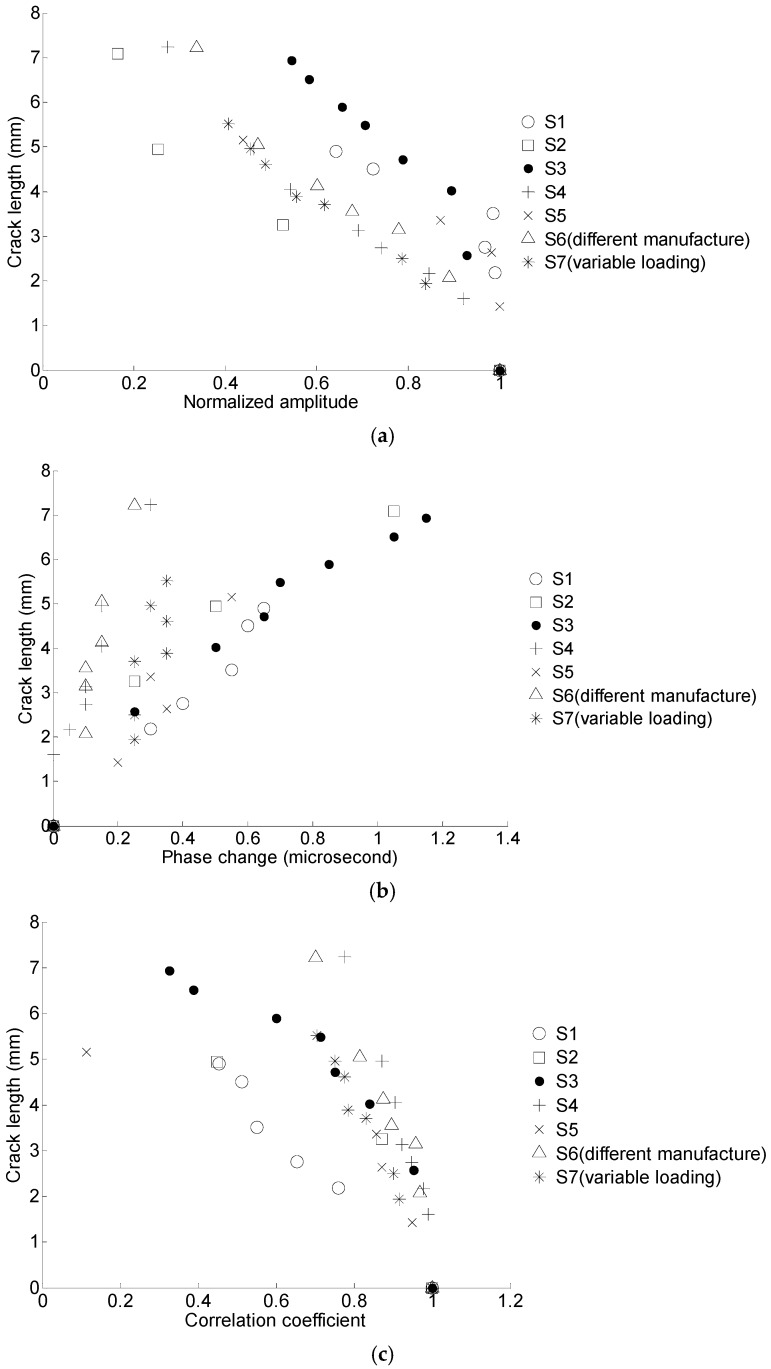
Crack length versus the damage sensitive features for seven specimens. (**a**) Crack length versus the normalized amplitude; (**b**) crack length versus the phase change; (**c**) crack length versus the correlation coefficient.

For the lap joint components fatigue test, the excitation frequency of the Lamb wave is 0.2 MHz. Similar to the coupon test, a symmetrical Hamming-windowed sinusoidal tone bust with 3.5 cycles is used as the excitation. Detailed information regarding the lap joint components fatigue test can be found in reference [14]. A total of seven specimens numbered as S1–S7 are used for the fatigue test. In order to verify the robustness of the proposed method for crack quantification, specimens from different manufactures with different loading spectra are used for fatigue testing. The loading spectrum for specimens S1–S6 is constant. A block loading is used for S7. Figure 18 shows the constant and variable amplitude loading spectra. Specimen S6 is made from a different manufacture with the same material and geometry. Similar to the coupon test, normalized amplitude, phase change, and correlation coefficient are extracted as the damage sensitive features. The proposed damage sensitive features are extracted for all seven tested specimens, as shown in Figure 19. The data of lap joint specimens exhibits a larger variation comparing with the coupon test due to the complexity in crack orientations, boundary conditions, and manufacturing uncertainty. 

### 4.2. Crack Quantificaiton Using GA Based LS-SVM

**Figure 20:**
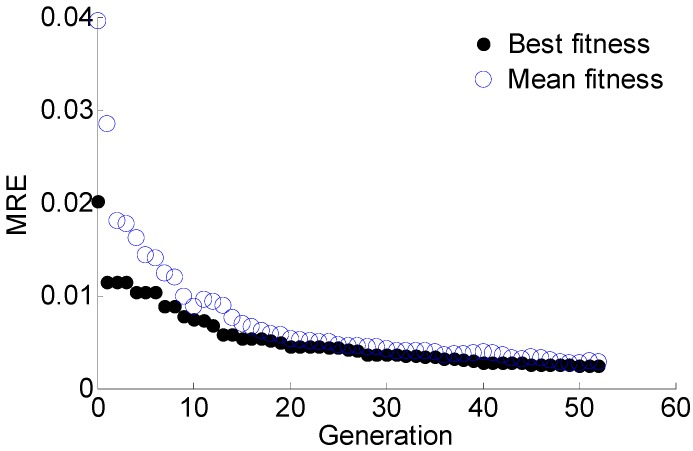
The optimization process using GA for model parameters.

The damage sensitive features data and corresponding crack size of specimens S2, S3, S4 are used to train the GA-based LS-SVM, and the data of specimens S1 and S5 are used to validate the trained model. The regularization parameter *γ* and the RBF parameter *σ* are optimized using GA with the objective of minimizing the MRE. The optimal target value MRE converges to 0.34% with the optimal results of *γ* = 17.0316 and *σ*^2^ = 0.0195, as shown in Figure 20. 

**Figure 21:**
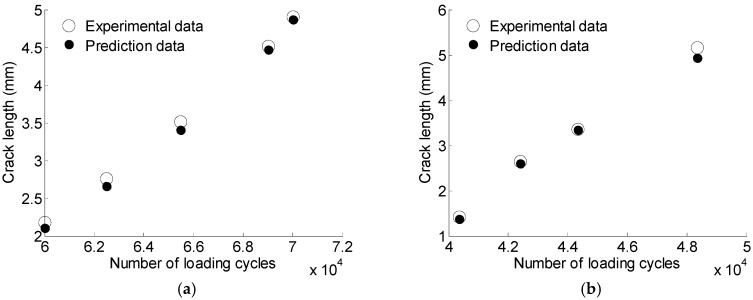
The prediction results for S1 and S5 using GA based LS-SVM. (**a**) S1; (**b**) S5.

Figure 21 shows the experimental data and the model predictions. It is observed that the proposed GA-based LS-SVM model can predict the crack size accurately.

**Figure 22:**
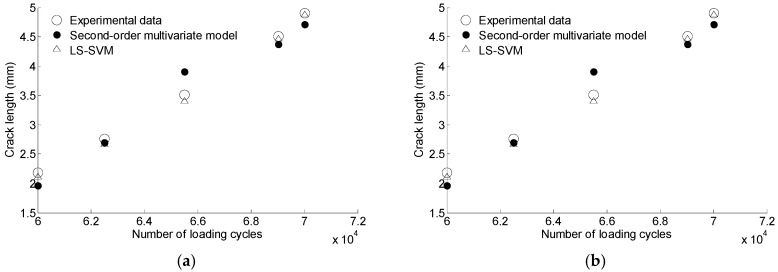
The comparison of prediction data based on second-order multivariate model and GA based LS-SVM. (**a**) S1; (**b**) S5.

The GA-based LS-SVM model is also compared with the second-order multivariate model proposed in reference [14]. The regression parameters of Equation (15) are shown in Table 7. The prediction results of the two methods are shown in Figure 22. It can be observed that the GA-based SVM generates more accurate results as the prediction points are closer to the actual points than that of the second-order multivariate model.

The comparison of the MRE results for GA-based LS-SVM and second-order multivariate model is shown in Table 8. GA-based LS-SVM yields smaller MREs compared with the second-order multivariate model, indicating that the proposed GA based LS-SVM can produce more accurate prediction results. 

### 4.3. Model Validation Using Different Loading and Manufacture

**Figure 23:**
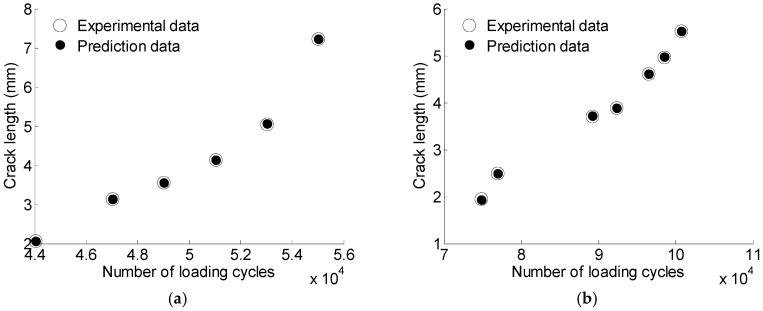
The prediction data for S6 and S7 using GA based LS-SVM. (**a**) S6; (**b**) S7.

To further verify the robustness of the proposed GA-based LS-SVM model for crack quantification, specimens with different loading spectrum (S7) and manufacture (S6) were investigated. The GA-based LS-SVM model obtained in Section 4.2 is used to predict the crack size for S6 and S7. The prediction results are shown in Figure 23. Table 9 presents the MRE results for S6 and S7.

The prediction results demonstrate that the GA-based LS-SVM model trained using specimens from one manufacture under constant loading can be used reliably and accurately to predict the crack size for specimens from another manufacture and specimens using a different loading. It indicates that the proposed method is robust against uncertainties associated with different loading cases and manufactures. 

### 4.4. Cross Validation

Cross validation is also used to validate the stability and robustness of the proposed GA based LS-SVM model under the lap joint components fatigue test. The data of S1–S5 are partitioned into two subsets. One is used as training data, which includes three specimens’ testing data, and the other is used for validation. There are in total 10 different partitions when choosing three from five, e.g., C(5,3) = 10. Except for the one combination presented above, the rest of the nine combinations are shown in Table 10. It was observed that the MRE for each result was small and stable.

In addition, the specimens with different loading spectrum (S7) and manufacture (S6) are investigated in the cross validation. The GA-based LS-SVM model trained by different training data in the cross validation is used to predict the crack size for S6 and S7. The MREs for S6 and S7 based on the prediction of different models are presented in Table 11.

## 5. Conclusions

This paper presented a method for crack size prediction using in-situ Lamb wave testing and GA-based LS-SVM. Three damage sensitive features are built based on physical interpretations. The three features, namely, normalized amplitude, phase change, and correlation coefficient, are extracted from the Lamb wave signal and used to develop LS-SVM. To enhance the robustness and training efficiency, a genetic algorithm is employed to obtain the optimal model parameters. A simple coupon test with six specimens was conducted to verify the effectiveness of the proposed method. The overall dataset is partitioned into two subsets, one for training and one for validation. The proposed GA-based LS-SVM is investigated thoroughly using the exhaustive cross validation technique. Furthermore, realistic lap joint components fatigue test data with naturally developed fatigue crack are used to validate the robustness and accuracy of the proposed GA-based LS-SVM model. Specimens from different manufactures and different loading spectra are designed to introduce inter-specimen uncertainty. The validation results indicate that the proposed method can predict the crack size reliably and accurately. Based on the current results, the following conclusions can be drawn.

(1) The proposed GA-based LS-SVM can accurately predict the crack size using Lamb wave data. An exhaustive cross validation is performed to investigate the performance of data-driven methods. Cross validation results indicate the proposed method is robust and is independent of the training dataset selection.
(2) The proposed GA-based LS-SVM can perform reliably under manufacturing and loading uncertainty. It provides a viable solution for structural health monitoring applications using the Lamb wave.

It is also worth mentioning that the crack lengths of all specimens (including the training set and validation set) in two cases are within the same size range. For the coupon test, all crack sizes are in the range of 0–30 mm. The range of crack size is 0–8 mm for the lap-joint case. Since the damage features are physics-based and the model incorporating the features is data-driven, the proposed method inherently has the limitation of extrapolation to the horizon out of the training size limit. Investigation on the extrapolation limit will be performed in a future study.

## Figures and Tables

**Table 1:** Mechanical properties of the testing specimen.

Material	Yield Strength (MPa)	Elastic Modulus (MPa)	*σ_u_* (MPa)
Al2043-T3	360	72,000	490

**Table 2:** Detailed information of the piezoelectric (PZT) (SM412) sensors.

Properties	Value
Diameter	7 mm
Thickness	0.2 mm
Density	7.80 g/cm^3^
Charge constant d_31_	−190 × 10^−12^ m/V
Charge constant d_33_	450 × 10^−12^ m/V
Relative dielectric constant	1580
Resonant frequency, *fr*	300 kHz ± 10 kHz

**Table 3:** The extracted damage sensitive features of specimen T1.

Crack Length (mm)	Normalized Amplitude	Phase Change	Correlation Coefficient
0	1	0	1
2	0.9616	0	0.9975
5	0.9657	0.1	0.9869
8	0.8620	0.2	0.9720
11	0.8051	0.2	0.9828
14	0.7753	0.3	0.9566
17	0.7931	0.4	0.9343
20	0.7647	0.5	0.9299
25	0.6799	0.5	0.8783
30	0.6592	0.6	0.8188

**Table 4:** The regression parameters for the second-order multivariate model.

A	α_1_	α_2_	α_3_	α_4_	α_5_	α_6_	α_7_	α_8_	α_9_
−353.51	306.87	442.27	439.06	−154.59	−269.38	−236.33	−45.06	−124.95	−77.03

**Table 5:** The comparison of predicted mean relative error (MREs).

Method	MRE (%)		
	T1	T2	T3
GA based LS-SVM	0.035	0.043	0.62
Second-order multivariate model	17.61	26.81	16.43

**Table 6:** The optimized parameters for different training data and the prediction results.

Model	Training Data	Optimized Model Parameters		Validation Data	MRE (%)		
		γ	*σ*^2^				
1	T3, T5, T6	11.2237	0.1799	T1, T2, T4	0.33	0.32	0.18
2	T3, T4, T6	4.3775	1.0658	T1, T2, T5	0.60	0.57	0.68
3	T3, T4, T5	11.4494	0.1834	T1, T2, T6	0.37	0.36	0.27
4	T2, T5, T6	12.0009	1.0371	T1, T3, T4	0.23	0.68	0.18
5	T2, T4, T6	7.5269	1.0264	T1, T3, T5	0.47	0.95	0.60
6	T2, T4, T5	11.2422	1.0411	T1, T3, T6	0.22	0.66	0.14
7	T2, T3, T6	9.1589	1.1383	T1, T4, T5	0.31	0.21	0.38
8	T2, T3, T5	6.6729	1.0555	T1, T4, T6	0.25	0.23	0.24
9	T2, T3, T4	5.5657	1.0893	T1, T5, T6	0.43	0.51	0.29
10	T1, T5, T6	13.9066	0.0060	T2, T3, T4	0.18	2.35	0.28
11	T1, T4, T6	9.2120	0.0448	T2, T3, T5	0.39	0.90	0.55
12	T1, T4, T5	8.9821	0.9917	T2, T3, T6	0.26	0.71	0.16
13	T1, T3, T6	11.1999	0.0359	T2, T4, T5	0.26	0.18	0.35
14	T1, T3, T5	5.8298	1.0529	T2, T4, T6	0.26	0.22	0.23
15	T1, T3, T4	7.8652	1.1164	T2, T5, T6	0.31	0.40	0.21
16	T1, T2, T6	8.4133	1.0438	T3, T4, T5	0.93	0.36	0.56
17	T1, T2, T5	5.0177	0.9988	T3, T4, T6	0.84	0.36	0.30
18	T1, T2, T4	12.2764	0.9849	T3, T5, T6	0.84	0.46	0.20
19	T1, T2, T3	10.2852	1.1510	T4, T5, T6	0.23	0.30	0.32

**Table 7:** The regression parameters for the second-order multivariate model.

A	*α*_1_	*α*_2_	*α*_3_	*α*_4_	*α*_5_	*α*_6_	*α*_7_	*α*_8_	*α*_9_
7.91	−2.76	−2.67	−9.41	0.52	−5.18	10.02	6.21	0.67	3.49

**Table 8:** The comparison of predicted MRE for different methods.

Method	MRE (%)	
	S1	S5
GA based LS-SVM	2.33	2.34
Second-order multivariate model	6.10	9.07

**Table 9:** The predicted MRE of S6 and S7.

Specimen	MRE (%)
S6	0.16
S7	0.19

**Table 10:** The optimized parameters for different training data and the prediction results.

Model	Training Data	Optimized Model Parameters		Validation Data	MRE (%)	
		γ	*σ*^2^			
1	S3, S4, S5	14.2149	0.0894	S1, S2	4.29	5.11
2	S2, S4, S5	12.3077	0.0065	S1, S3	6.62	6.46
3	S2, S3, S5	13.8345	0.1044	S1, S4	3.09	1.84
4	S1, S4, S5	11.7484	0.0464	S2, S3	6.00	3.78
5	S1, S3, S5	15.2365	0.3174	S2, S4	6.32	1.36
6	S1, S3, S4	15.7772	0.0188	S2, S5	4.23	4.63
7	S1, S2, S5	14.1009	0.0527	S3, S4	5.03	4.73
8	S1, S2, S4	14.7378	0.0765	S3, S5	3.85	4.05
9	S1, S2, S3	17.0275	0.0389	S4, S5	4.45	5.01

**Table 11:** The predicted MREs of S6 and S7 based on different training data.

Model	Training Data	MRE (%)	
		S6	S7
1	S3, S4, S5	0.39	0. 34
2	S2, S4, S5	2.68	3.78
3	S2, S3, S5	1.62	0. 93
4	S1, S4, S5	0.61	0.61
5	S1, S3, S5	1.20	0.96
6	S1, S3, S4	0. 80	0. 95
7	S1, S2, S5	4.01	4.23
8	S1, S2, S4	0. 54	0. 36
9	S1, S2, S3	3.19	2.57
